# *In vivo*-induced size transformation of cerium oxide nanoparticles in both lung and liver does not affect long-term hepatic accumulation following pulmonary exposure

**DOI:** 10.1371/journal.pone.0202477

**Published:** 2018-08-20

**Authors:** Justyna Modrzynska, Trine Berthing, Gitte Ravn-Haren, Kirsten Kling, Alicja Mortensen, Rie R. Rasmussen, Erik H. Larsen, Anne T. Saber, Ulla Vogel, Katrin Loeschner

**Affiliations:** 1 National Food Institute, Technical University of Denmark, Kongens Lyngby, Denmark; 2 The National Research Centre for the Working Environment, Copenhagen, Denmark; 3 Department of Micro- and Nanotechnology, Technical University of Denmark, Kongens Lyngby, Denmark; Brandeis University, UNITED STATES

## Abstract

Recent findings show that cerium oxide (CeO_2_) nanoparticles may undergo *in vivo*-induced size transformation with the formation of smaller particles that could result in a higher translocation following pulmonary exposure compared to virtually insoluble particles, like titanium dioxide (TiO_2_). Therefore, we compared liver deposition of CeO_2_ and TiO_2_ nanoparticles of similar primary sizes 1, 28 or 180 days after intratracheal instillation of 162 μg of NPs in female C57BL/6 mice. Mice exposed to 162 μg CeO_2_ or TiO_2_ nanoparticles by intravenous injection or oral gavage were included as reference groups to assess the amount of NPs that reach the liver bypassing the lungs and the translocation of NPs from the gastrointestinal tract to the liver, respectively. Pulmonary deposited CeO_2_ nanoparticles were detected in the liver 28 and 180 days post-exposure and TiO_2_ nanoparticles 180 days post-exposure as determined by darkfield imaging and by the quantification of Ce and Ti mass concentration by inductively coupled plasma-mass spectrometry (ICP-MS). Ce and Ti concentrations increased over time and 180 days post-exposure the translocation to the liver was 2.87 ± 3.37% and 1.24 ± 1.98% of the initial pulmonary dose, respectively. Single particle ICP-MS showed that the size of CeO_2_ nanoparticles in both lung and liver tissue decreased over time. No nanoparticles were detected in the liver following oral gavage. Our results suggest that pulmonary deposited CeO_2_ and TiO_2_ nanoparticles translocate to the liver with similar calculated translocation rates despite their different chemical composition and shape. The observed particle size distributions of CeO_2_ nanoparticles indicate *in vivo* processing over time both in lung and liver. The fact that no particles were detected in the liver following oral exposure showed that direct translocation of nanoparticles from lung to the systemic circulation was the most important route of translocation for pulmonary deposited particles.

## Introduction

Unique properties exhibited by nanoparticles (NPs) such as small size, large surface area and high reactivity as compared to fine particles or bulk material has led to the development of a wide range of industrial applications of NPs. NPs can be used in various day-to-day consumer products including cosmetics, electronics, sporting goods, paints, lacquers and tires. They could also serve as food additives and cosmetics Therefore, several portals of entry and different target tissues exist [1–4]. Understanding the deposition fate of NPs after administration to the body is a crucial part of hazard evaluation of nanomaterials.

Both cerium oxide (CeO_2_) and titanium dioxide (TiO_2_) NPs have versatile applications and are widely used in everyday products. CeO_2_ can be used as an abrasive agent for chemical-mechanical planarization of advanced integrated circuits [5], polishing agent for glass mirrors, television tubes and ophthalmic lenses [6], oxygen sensor [7], diesel fuel catalyst to improve combustion and therefore reduce consumption of the fuels [6] and in solid oxide fuel cells [8]. TiO_2_ is widely used as white pigment due to its brightness and high refractive index [9] and is therefore utilized in paints, coatings, cosmetics (sunscreen, toothpaste) and in pharmaceuticals [10]. Moreover, it has been approved as a food additive in Europe (also known as E171) used in candy and chewing gum [11].

Inhalation is the most relevant exposure route for occupational exposure to NPs. It has been previously observed that particles deposited in the lungs after inhalation or intratracheal instillation are cleared from the respiratory tract by various clearance mechanisms and various routes. Inhaled nanosized particles, deposited preferentially in the alveolar region, are cleared mainly by macrophage-mediated phagocytosis. Internalized particles gradually moved toward the mucociliary escalator in the upper airways [12]. Once the particles reach the bronchi, they are transported up into the pharynx and swallowed, causing gastrointestinal tract (GIT) exposure [2,13–16]. Nonetheless, the uptake of NPs by the GIT has been shown to be very low [9,15,17–19]. Inhaled NPs may also migrate across the alveolar epithelium, through interstitium, access systemic circulation directly or via lymphatic vessels and accumulate in the liver and spleen [20–25]. Inconsistent results regarding the fraction of ultrafine particles (UFP) that undergo extrapulmonary translocation and further deposition in the secondary organs have been reported [14,25–27]. The degree of translocation is influenced by various physicochemical properties like size, chemical composition, shape and electrical charge [14,28].

The liver, among other reticuloendothelial organs, seems to be the major organ for accumulation of insoluble particles such as metal NPs after accessing systemic circulation [29–33]. Translocated NPs reach the liver, and they have been shown to accumulate in the Kupffer cells, which are specialized macrophages located in the liver [29,34,35]. The Kupffer cells constitute the first line of the defense against xenobiotics. The clearance rate of NPs from Kupffer cells is very slow. Thus, 90% of an injected gold NP (40 nm) dose was still detected in the liver tissue 6 months after exposure [36]. Previous studies indicate that pulmonary exposure to reactive NPs is associated with genotoxic effect observed in the liver tissue [13,37–39].

Graham and colleagues [40] recently showed that hydro-thermally derived CeO_2_ NPs undergo *in vivo* processing in the liver tissue over time. The originally cube-shaped CeO_2_ NPs became highly fragmented and rounded along their edges ninety days after exposure. No NP transformation was observed thirty days after exposure. Additionally, the accumulation of 1–3 nm crystallites in close proximity to the parental CeO_2_ NPs was observed. Dissolution of high-energy edge sites was suggested as the underlying mechanism.

The aim of the present study was to evaluate extrapulmonary translocation of lung-deposited CeO_2_ and TiO_2_ and to assess whether the partial solubility of CeO_2_ affect hepatic accumulation. We also wanted to determine size distribution changes of long-term deposited particles in the lung and liver tissue.

## Materials and methods

### Preparation of NP suspensions and characterization of exposure media

Powdered cerium dioxide (CeO_2_) was provided by Degussa-Quimidroga and powdered titanium dioxide (TiO_2_) was provided by NanoAmor (Nanostructured & Amorphous Materials). NP suspensions of 3.24 mg/ml were prepared in 2% mouse serum as described in [41,42]. TiO_2_ NPs have been shown to induce similar inflammatory responses when dispersed in nanopure water or 2% serum [43], suggesting that the serum was not immunogenic in the present experimental set-up. 2% serum was chosen since dispersion in this vehicle resulted in stable dispersion of nanosized aggregates. In short, stock suspensions of 3.24 mg/ml alongside control vehicle were sonicated for 15 min on ice, to prevent sample overheating, using a probe sonicator operating at 100 W/22.5 kHz (Microson ultrasonic cell disruptor, XL-2000 Microson™, Qsonica, LLC., equipped with disruptor horn with a diameter of 3.2 mm and maximum peak-to-peak amplitude of 180 μm). The size distribution of each produced NP suspension was determined by dynamic light scattering (Zetasizer Nano-ZS, Malvern Instruments, UK) as described [38,44]. The refractive index and the index of absorption for the test materials were: 2.2 and 0.1, respectively, for CeO_2_, 2.903 and 0.1, respectively, for TiO_2._ Optical data for water were used for the control vehicle.

### Preparation and analysis of electron microscopy samples

Samples were dispersed on TEM (Transmission Electron Microscope) grids covered with holy carbon foil according to a standard protocol for diameter and morphological analyses of nanomaterial in SEM (Scanning Electron Microscope) and TEM. 0.2 mg of CeO_2_ and 0.5 mg of TiO_2_ were dispersed in 8 ml of N-methyl 2-pyrrolidone and incubated for 10 min in an ultrasound bath. The dispersion was sonicated using a probe sonicator with a 13 mm probe tip for 10 min at 10% amplitude on ice. After sonication, 10 μL of sample was dispersed onto a TEM-grid. The samples were dried in a vacuum chamber for 5 min. Afterwards samples were further dried under red light (warming lamp) for 24 h.

Images were recorded at the ZEISS facility in Oberkochen, Germany by curtesy of Dr. Xiong Liu, using a GeminiSEM 300 equipped with a STEM detector (ZEISS, Oberkochen, Germany). Plasma cleaning was applied to the samples inside the microscope chamber, to derive a better image quality.

### Animal study

216 young adult C57BL/6 (B6JBOM-F) female mice were purchased from Taconic (Ry, Denmark) at 6 weeks of age and body weight (bw) of 17.5 ± 0.9 g (mean ± SD). Mice were acclimatized for 2 weeks before the beginning of the experiment. Upon delivery mice were randomly divided into experimental groups and control groups (n = 9) and housed in polypropylene cages (Type III with Tapvei bedding and enrichment–nesting material and den), 5 or 4 mice in each, under the following conditions: 12 h light/12 h dark cycle, room temperature of 22°C ± 1° and the relative humidity of 55% ± 5. Throughout the whole experiment mice were given a standard pellet diet (Altromin No. 1324) and citric acid acidified tap water (to avoid microbiological contamination of drinking water), both *ad libitum*. All mice were weighted weekly and food intake was noted. For 6 months exposure groups, bw was recorded once in 2 weeks intervals after day 28. Mice were observed daily for any sign of abnormalities in their clinical appearance.

Mice were dosed a single dose of 162 μg of CeO_2_ or TiO_2_ NPs in 50 μl of 2% serum in nanopure water by intratracheal instillation (IT), oral gavage (PO) or intravenous injection (IV). Control mice received 50 μl of 2% serum. The dose corresponds to the pulmonary deposition during 13 8-h working days at the Danish occupational exposure limit of 10 mg/m^3^ for TiO_2_ assuming 9% alveolar deposition [45,46]. Prior to the administration of NP suspension by either intravenous injection or intratracheal instillation animals were anaesthetized by subcutaneous injection in the neck with 0.5 ml/100g of Hypnorm^®^ (Fentanyl citrate 0.315 mg/ml and Fluanisone 10 mg/ml, Nomeco) and Dormicum^®^ (Midazolam 5 mg/ml, Roche) diluted 1:1 by volume in sterile water and mixed. Orally administered mice were not anaesthetized during dosing. Intratracheal instillation was performed as previously described [46]. In brief, mice were placed on a 40° slope (upside down, with the head towards the floor) and a diode lamp was located on the larynx for a better visualization. A small spatula was used to press the tongue towards the lower jaw and the 22 GA BD Insyte catheters (Becton Dickinson, Utah, USA) with a shortened needle was used to intubate the trachea. A volume of 50 μl followed by 200 μl air in a 250 μl SGE glass syringe (250F-LT-GT, MicroLab, Aarhus, Denmark) was instilled. In order not to block the airways and to assure that the NP suspension maintains in the lung, mice were placed back into a vertical position with the head up as soon as the catheter was removed. For oral gavage, mice were gently restrained in a vertical position to immobilize the head and the gavage needle (0.9 x 38 mm, NOVA-SCB, Sweden) was inserted into the esophagus and further toward the stomach. For intravenous injection anaesthetized mice were restrained in a Plexiglas restraining tube. A tail-vein injection was performed with a 0.4 × 20 mm needle (Terumo Europe, n.v. 3001, Leuven, Belgium). After intratracheal instillation, gavage and intravenous injection animals were transferred back to their cages, heated with a heating lamp and/or warming blanket and monitored until they fully recovered from anesthesia. The animals did not show any adverse effects caused by the administration procedure. Ethical approval was given by the Danish Animal Experiments Inspectorate (Permission 2012-15-2934-00089 C6) and the specific experiment was approved and overseen by the Animal Welfare Committee for Animal Care and Use of the National Food Institute, Technical University of Denmark.

### Necropsy and organ collection

After the exposure period– 1 day, 28 days or 180 days, animals were weighted and prepared for necropsy. Mice were terminated by the overdose of Hypnorm–Dormicum mixture (0.5–0.7 ml/100 g). The mice abdomen and thorax were opened and all macroscopic abnormalities were noted in the autopsy report. Pieces of liver and lung (right caudal lobe) tissue for further ICP-MS analysis were collected and snapped frozen in cryotubes (NUNC) in liquid nitrogen and stored at—80°C until used. A piece of liver tissue was fixated in 4% neutral buffered formalin for 24 hours and embedded in paraffin blocks on the next day. Lungs were removed intact, filled with formalin and submerged in 4% neutral buffered formalin. Liver and lungs tissue sections of 3 μm thickness were cut and stained with hematoxylin and eosin for further light and darkfield microscopy.

### Enhanced darkfield microscopy

Cytoviva enhanced darkfield hyperspectral system (Auburn, AL, USA) was used to detect particles in liver and lung, by scanning histological sections at 40x magnification in enhanced darkfield mode. Images were acquired at 40x and 100x using an Olympus BX 43 microscope with a Qimaging Retiga4000R camera. Uneven illumination in brightfield images was corrected using ImageJ [47] and the Calculator Plus plugin via the formula: Corrected image = (image / background) * 255. The background image was a maximum projection of 3 background brightfield images without tissue.

### Determination of mass concentration of Ce and Ti in mouse organs by ICP-MS

The total mass concentration of cerium (Ce) and titanium (Ti) in animal tissues (liver and lung) was determined by inductively coupled plasma-mass spectrometry (ICP-MS). Tissue samples (100 mg for liver and 40 mg for lung) were taken from the same part of the lungs and livers from each animal. Ultrapure water (18.2 mΩ·cm at 21.5°C) was obtained from a Millipore Element apparatus (Millipore, Milford, MA, USA) and used throughout the work.

For analysis of Ce by ICP-MS, approximately 100 mg of the liver tissue was thawed, exactly weighed, mixed with 1.5 ml of ultrapure water and homogenized using an Ultra Turrax homogenizer (DI 25 Basic, Ika-Werke, Staufen, Germany) for 3 min at 8000 rpm. For Ti analysis Ceramic Bead Tubes (⌀ 1.4 mm, made of zirconium oxide, MoBio, Denmark) were used for liver tissue homogenization to avoid potential release of Ti-containing particles from the Ultra Turrax homogenizer. Approximately 100 mg of the liver tissue was thawed, exactly weighed, mixed with 1.5 ml of ultrapure water and ceramic beads and subsequently homogenized for 3 min using a Tissue Lyser (Qiagen, manufactured by Retsch). A volume of tissue homogenate corresponding to 40 mg of tissue was transferred to disposable standard glass vials (Wheaton® 15x46 mm, cap 13–425) and subsequently digested in a mixture of 0.250 ml concentrated nitric acid (68% HNO_3_, PlasmaPure, SCP Science, Quebec, Canada) and 0.125 ml hydrogen peroxide (Suprapur, Merck, Darmstadt, Germany) in a microwave oven (Multiwave 3000, Anton Paar GmbH, Graz, Austria) equipped with a 64MG5 rotor at elevated temperature and pressure (~ 140°C; max. 20 bar). After digestion, ultrapure water was added to a final sample weight of 3 g. Samples were further diluted 10- to 1000-fold with ultrapure water prior to analysis. The rest of the Ce and Ti homogenate was stored at -20°C for single particle ICP-MS analysis. Approximately 40 mg of the lung tissue was prepared as described above excluding the homogenization procedure.

The Ce mass concentration in the tissues was determined by using an iCAP Q ICP-MS instrument (Thermo Fisher Scientific GmbH, Bremen, Germany) equipped with an ASX-520 autosampler (Cetac Technologies, Omaha, USA). Typical instrumental settings for the analysis are given in Table 1. The concentration of Ce was quantified against an external calibration curve prepared in 2% nitric acid from a Ce stock solution of 1000 μg/ml (SCP Science, Quebec, Canada). To reduce carry-over, careful rinsing with 2% nitric acid between samples was performed.

**Table 1:** Instrumental settings for ICP-MS analysis.

Parameter (unit)	Ce analysis	CeO_2_ single particle analysis	Ti analysis
RF power (W)	1550	1550	1550
Plasma gas flow rate (l/min)	14	14	15
Nebulizer gas flow rate (ml/min)	~1.1	~1.1	~1.1
Auxiliary gas flow rate (ml/min)	~0.8	~0.8	~0.1
Cell gas flow rate (ml/min)	n/a	n/a	2 ml/min NH_3_/He (10/90) + 1 ml/min He
Monitored isotopes (*m/z*)	^140^Ce	^140^Ce, ^197^Au	Q1 → Q2 masses: 48→ 150 (Ti); 45 → 130 (Sc)
Dwell time (ms)	10	3	100
Nebulizer type	Low-flow concentric nebulizer	Low-flow concentric nebulizer	Agilent MicroFlow (model no. G3139A-100)
Spray chamber type	Cyclonic, Peltier-cooled (quartz)	Cyclonic, Peltier-cooled (quartz)	Scott double-pass, Peltier-cooled (quartz)

The Ti mass concentration in the tissues was determined by using an Agilent 8800 Triple Quadrupole ICP-MS instrument (Agilent Technologies, California, USA) equipped with an ASX-520 autosampler (Cetac Technologies, Omaha, USA). Typical instrumental settings for the analysis are given in Table 1. The concentration of Ti was quantified against an external calibration curve prepared in 2% nitric acid from Ti stock solution of 1.000 μg/ml (SCP Science, Quebec, Canada) and with scandium (Sc) as an internal standard (stock solution of 1.000 μg/ml, SCP Science, Quebec, Canada). To reduce carry-over, careful rinsing with 2% nitric acid between samples was performed.

Blank samples, laboratory duplicates and spiked samples were included in all analyses for quality control. No suitable certified reference materials for CeO_2_ or TiO_2_ were available. The limit of detection (LOD) in the liver tissue was the range of 39–112 ng/g for Ce and between 539–852 ng/g for Ti. LOD in the lung tissue was estimated to be between 8–14 ng/g for Ce and between 742 and 2192 ng/g for Ti. The LOD in tissues varied for the different sample types due to different sample intake in the digestion step and the sample dilution prior to analysis.

### Determination of size distribution of CeO_2_ NPs in mouse organs by spICP-MS

A previously developed enzymatic digestion procedure was modified and applied [48]. Briefly, a volume of liver homogenate which contained 25 mg of liver tissue or ~ 25 mg of unhomogenized lung tissue was mixed with 3 ml of 3 mg/ml Proteinase K solution in 50 mM ammonium bicarbonate buffer at pH 7.4, containing 5 mg/ml SDS and 0.2 mg/ml NaN_3_ as antimicrobial agent and 1 ml of ultrapure water. The mixture was incubated over night at 40°C in a water bath under continuous stirring. As a blank sample, unexposed liver or lung tissue was included. Prior to the spICP-MS measurement, samples were further diluted between 100- and 50.000-fold with ultrapure water, depending on the Ce mass concentration measured by conventional ICP-MS.

The iCAP Q ICP-MS instrument was used for spICP-MS analysis. Instrument settings are given in Table 1. The peristaltic pump was set to 40 rpm for all experiments, which corresponded to a sample flow rate of approximately 0.4 ml/min. This value was accurately determined daily by weighing the amount of water that was delivered by the peristaltic pump for 1 min. Ce mass calibration was achieved by analysis of a blank and five ionic Ce standards ranging from 0.2 to 5.0 ng/l diluted from a certified solution in 100x diluted enzymatically digested liver tissue. The ^140^Ce intensity for each solution was averaged from the entire length of the analysis (180 s). To evaluate for possible instrumental drift over time the standards were analyzed at the beginning and the end of the analysis sequence. The nebulization efficiency of the liquid samples through the sample introduction system was determined according to the “particle frequency” method [49] by the measurement of reference gold nanoparticle (AuNP) suspension (RM 8013, National Institute of Standards and Technology, Gaithersburg (NIST), MD, USA) with a known average particle diameter of 56 nm (based on the measurements by transmission electron microscopy provided by NIST) and a gold mass concentration of 51.86 ± 0.64 μg/g (information value provided by NIST), diluted 10^6^-times with ultrapure water. The nebulization efficiency was calculated as the percentage of all Au NPs detected by spICP-MS versus the theoretical (calculated) particle number in the introduced sample volume, derived from the TEM-based average size, the measured uptake rate and time of introduction of the sample suspension into the ICP-MS instrument.

For each sample the ^140^Ce signal intensity was continuously monitored for 60 s or 180 s which corresponded to 20,000 or 60,000 data points, respectively, when using 3 ms dwell time. Following the analysis of each sample, ultrapure water was analyzed to illustrate if carry-over from the previous measurement could be excluded. Intensity data were recorded by the ICP-MS software and exported to Microsoft Excel 2010 for Windows (Microsoft Inc., WA, USA) for further processing. Raw signal intensity data were plotted (in counts per second) versus number of events to create a signal distribution histogram. Very low signal intensities were considered instrument background or for slightly higher intensity values dissolved cerium or cerium clusters. An iterative algorithm was applied where “particle events” were distinguished as outliers from the background/dissolved ion signal if the measured intensity was more than five times the standard deviation of the whole data set as described by [50]. For each “particle event”, the signal intensity was converted to Ce mass (per particle) based on the slope of the calibration curve obtained with the ionic Ce standards multiplied with dwell time, sample flow rate and nebulization efficiency. Finally, the Ce mass was converted to particle diameter by assuming spherical particles of CeO_2_ (fraction of Ce 0.53) with densities of 7.132 g/cm^3^.

### Statistical analysis

All presented values are expressed as mean ± standard deviation of the mean (SD) unless stated differently. One-way or two-way analysis of variance (ANOVA) was used to analyze the data sets. In order to fulfil the normality and variance homogeneity criteria some variables were logarithmically transformed. Non-normally distributed data were ranked before applying nonparametric one-way or two-way ANOVA analysis. If the statistical significance was reached in the ANOVA analysis, Tukey *post-hoc* multiple comparison test was used to test the differences between the test groups. P-value of 0.05 was considered significant. All statistical analyses were calculated using SAS 9.4 statistical software (SAS Institute Inc., Cary, NC, USA).

## Results

### Mortality

After administration of NPs mice were monitored daily for any signs of sickness or injury, abnormalities in their general appearance as well as changes in their behavior. Among 216 mice used in the experiment, 3 unexpected deaths were reported during the up to 6 months long post exposure period. These were one mouse from the PO 6 months CeO_2_ group, one mouse from IV 28 days control group, one mouse was from IV 6 months TiO_2_ group. The causes of death were not established and the mice were excluded from the experiment.

### Physicochemical characterization of nanomaterials

**Fig 1:**
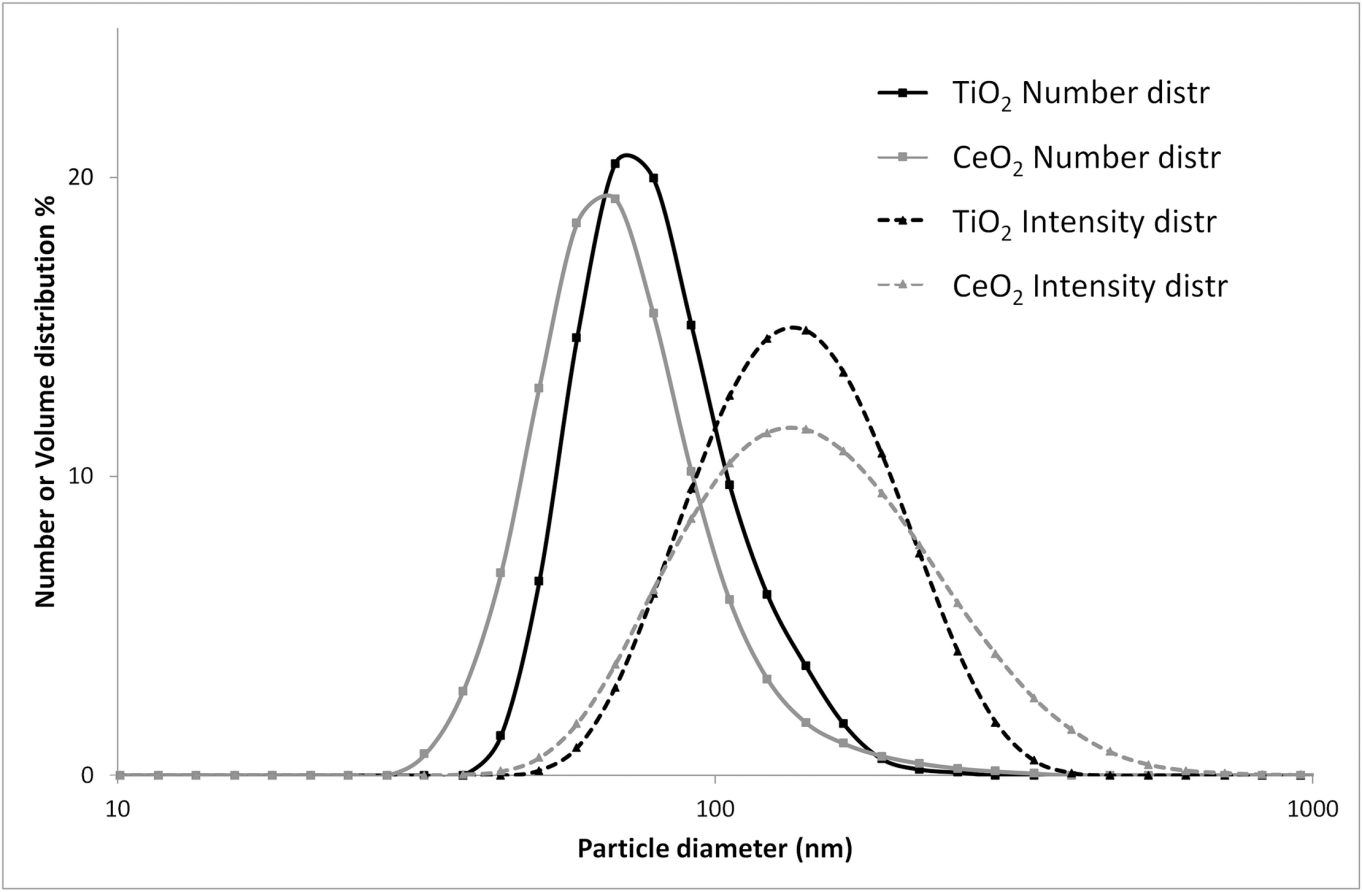
Size distributions of CeO_2_ and TiO_2_ particle suspensions obtained by DLS. CeO_2_ and TiO_2_ particles were suspended in 2% serum in nanopure water. Intensity- and number-based size distributions are presented. The figure was adapted from [39].

The key physicochemical parameters of the studied nanomaterials have been previously published [51–53] and are summarized in Table 2. Agglomerate size of particle suspensions was determined using dynamic light scattering (DLS). The hydrodynamic number-based size distribution showed a single peak with the average diameter below 100 nm for both CeO_2_ and TiO_2_ NP suspensions (Fig 1). Median particle diameters measured for CeO_2_ and TiO_2_ were 79 nm and 68 nm, respectively. Z-average and polydispersity index were 159.7 and 0.216, respectively, for CeO_2_ and 125.9 and 0.122, respectively, for TiO_2._ For CeO_2_, the size distribution of the particle suspension was additionally determined by spICP-MS (S1 Fig). The median diameter was around 50 nm. However, when the suspension was enzymatically treated, the median diameter decreased to around 35 nm. TiO_2_ NPs were not characterized by spICP-MS due to the relatively high size LOD of 50–60 nm for this type of NPs.

**Table 2:** Key physicochemical parameters of the tested nanomaterials.

	CeO_2_	TiO_2_ (rutile)
Source	Degussa/Quimidroga^a^	NanoAmor^b^
Product form	Powder	Powder
Primary particle size	13.0 ± 12.1 nm^a^	10.5 nm^b^
Specific surface area	56.7 m^2^/g^a^	139.1 m^2^/g^b^
Particle density	7.29 g/cm^3^^a^	4.23 g/cm^3^^c^

^a^Levin et al. J Nanoparticle Res. 2015;17:1–13

^b^Halappanavar et al. Environ Mol Mutagen. 2015;56:245–264

^c^Lide DR. CRC Handbook of Chemistry and Physics 84th ed. Boca Raton: CRC Press LLC; 2003.

**Fig 2:**
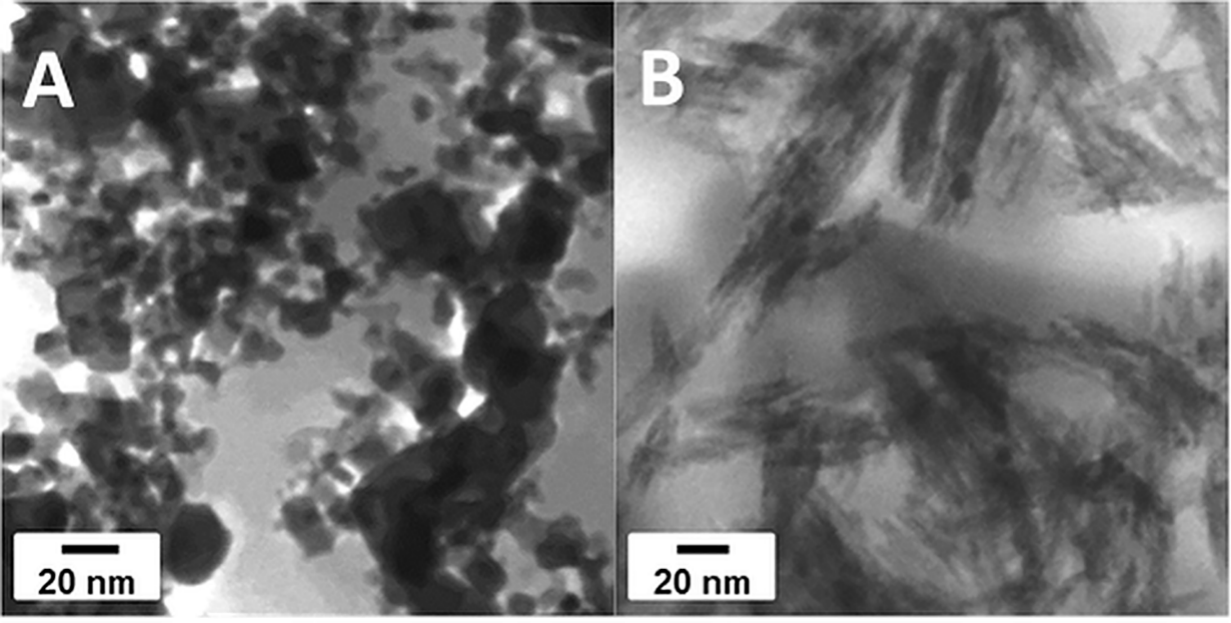
STEM images. CeO_2_ NPs (A) and TiO_2_ NPs (B).

In STEM investigations, the CeO_2_ NPs appeared as aggregates of primary crystals (Fig 2A). Images taken at high magnification in STEM mode (S2 Fig) showed that the size of primary particles varies from few nm up to several tens of nm. The aggregate size cannot be determined from the images obtained. Particles regardless of size appeared cubic (fluorite structure, space group Fm3m) however, the crystalline structure and phase of particles was not investigated in the scope of this study. TiO_2_ NPs appeared as aggregates of primary crystals (Fig 2B). Images taken at high magnification in STEM mode (results not shown) showed that the primary particles were nanorods with a length of 50 to 60 nm and a diameter of 10 to 15 nm.

### Brightfield and enhanced darkfield microscopy of lung and liver tissues

**Fig 3:**
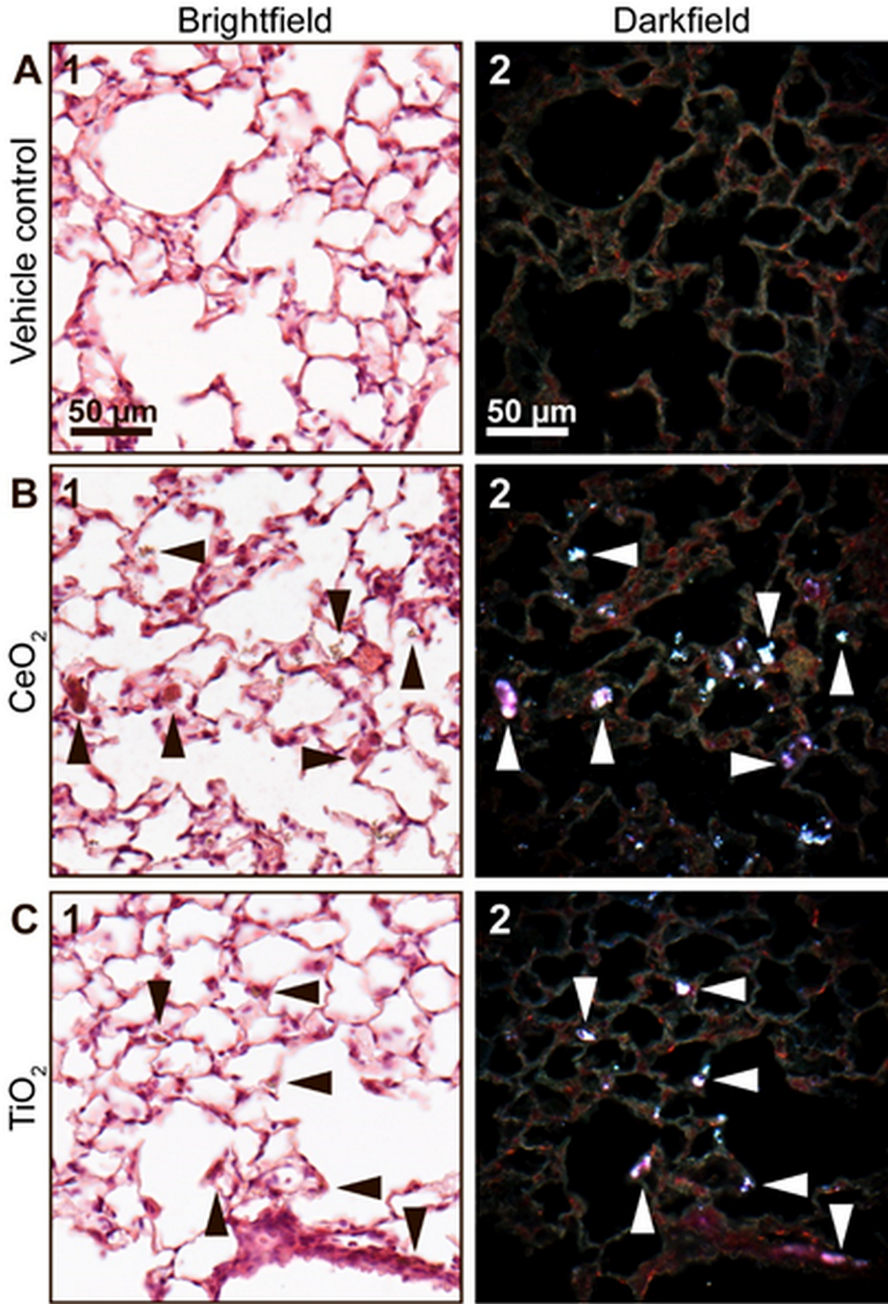
Brightfield (1) and enhanced darkfield (2) microscopy images of H&E stained lung tissue. (A) From intratracheally instilled mice that received a control vehicle, (B) 162 μg/animal of CeO_2_ or (C) TiO_2_ NPs at 180 days post exposure. In the lung sections from mice exposed to CeO_2_ and TiO_2_ light scattering aggregates of foreign material were observed using enhanced darkfield microscopy (B2 and C2, respectively, white arrowheads). The aggregates exhibited a brownish appearance in brightfield (B1 and C1, black arrowheads).

**Fig 4:**
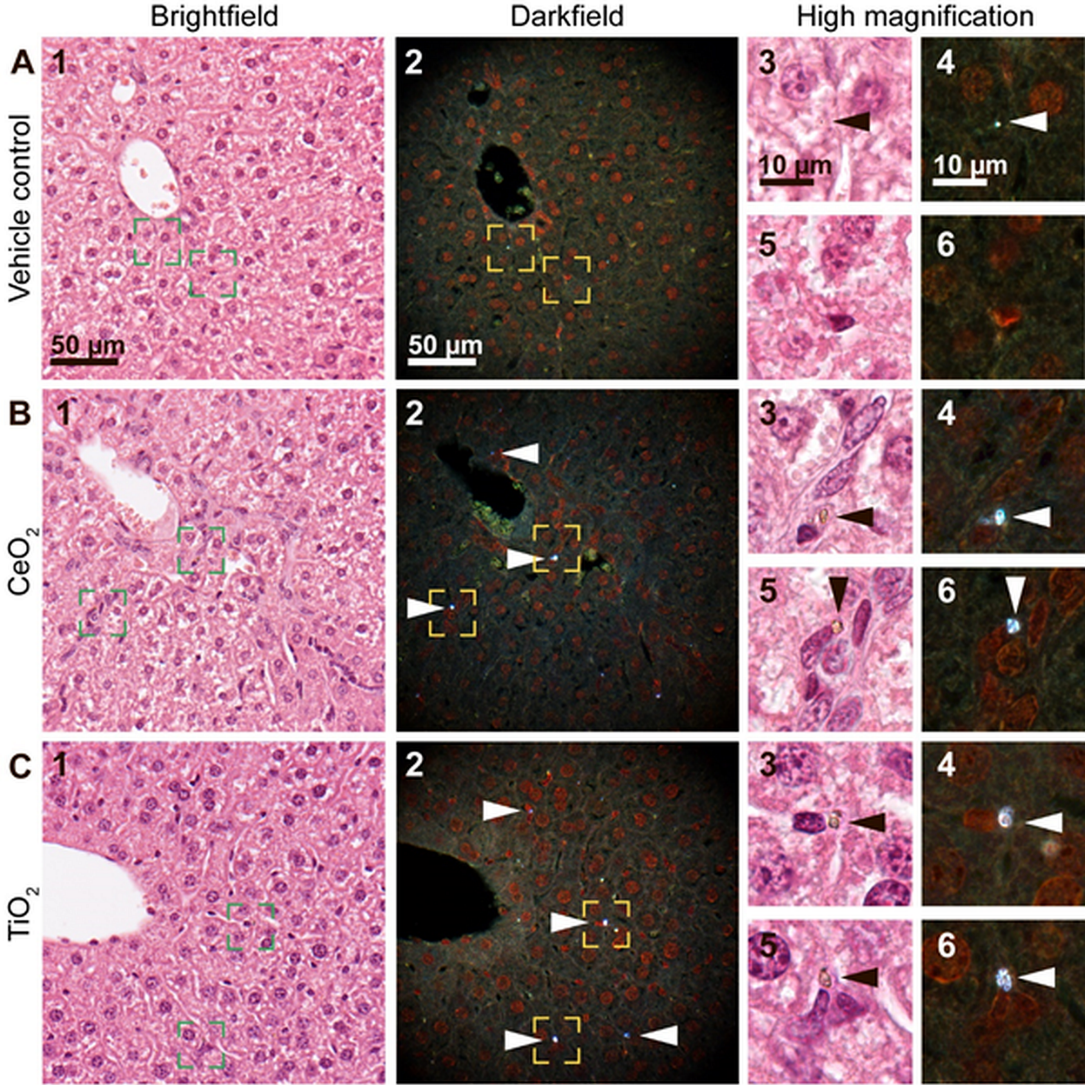
Brightfield (1) and enhanced darkfield (2) microscopy images of H&E stained liver tissue. (A) From intratracheally instilled mice that received a control vehicle, (B) 162 μg/animal of CeO_2_ or (C) or TiO_2_ NPs at 180 days post-exposure. In the liver sections from mice exposed to CeO_2_ and TiO_2_ foreign material aggregates were observed using enhanced darkfield microscopy, mainly in sinusoids and often close to small nuclei (B and C, respectively, 2, 4 and 6, white arrowheads). The aggregates were not detectable in brightfield at 40x magnification (B1, C1), but exhibited a brownish appearance at 100x magnification (B and C, 3 and 5, black arrowheads). Appearance of a typical artefact is shown (A, 3–4, arrowheads). Panels 3–6 correspond to the rectangular zones in panel 1–2 captured at higher magnification.

Figs 3 and 4 show lung and liver sections, respectively, from mice intratracheally instilled with a control vehicle (2% serum) or 162 μg/animal of CeO_2_ or TiO_2_ 180 days post exposure. Lung sections from mice exposed to CeO_2_ and TiO_2_ revealed presence of foreign material aggregates in the pulmonary region, thus documenting successful pulmonary dosing. The aggregates showed intense light scattering in enhanced darkfield (Fig 3B2 and 3C2, white arrowheads) and had a brownish appearance in brightfield (Fig 3B1 and 3C1, black arrowheads). In the two exposure groups, the foreign material was predominantly found in the alveolar region, retained in macrophages, in the interstitium, and in the alveolar lumen (Fig 3B and 3C).

Sections of liver tissue were scanned for presence of foreign material. Liver sections from mice exposed to CeO_2_ and TiO_2_ by intratracheal instillation revealed the presence of light scattering aggregates of foreign material that were also visible in brightfield at high magnification. The foreign material was predominantly found in sinusoids and there often close to nuclei, which based on shape and location could be Kupffer cell nuclei (Fig 4B and 4C, white and black arrowheads). In all groups, including vehicle controls, highly scattering material with a different appearance (intensity, size or color) was occasionally found (example in Fig 4A, arrowheads). The localization indicated artefacts from tissue preparation.

Following oral gavage, neither CeO_2_ nor TiO_2_ NPs could be detected in liver tissue (S3 Fig).

### Biodistribution of NPs in the liver and lung tissue

**Fig 5:**
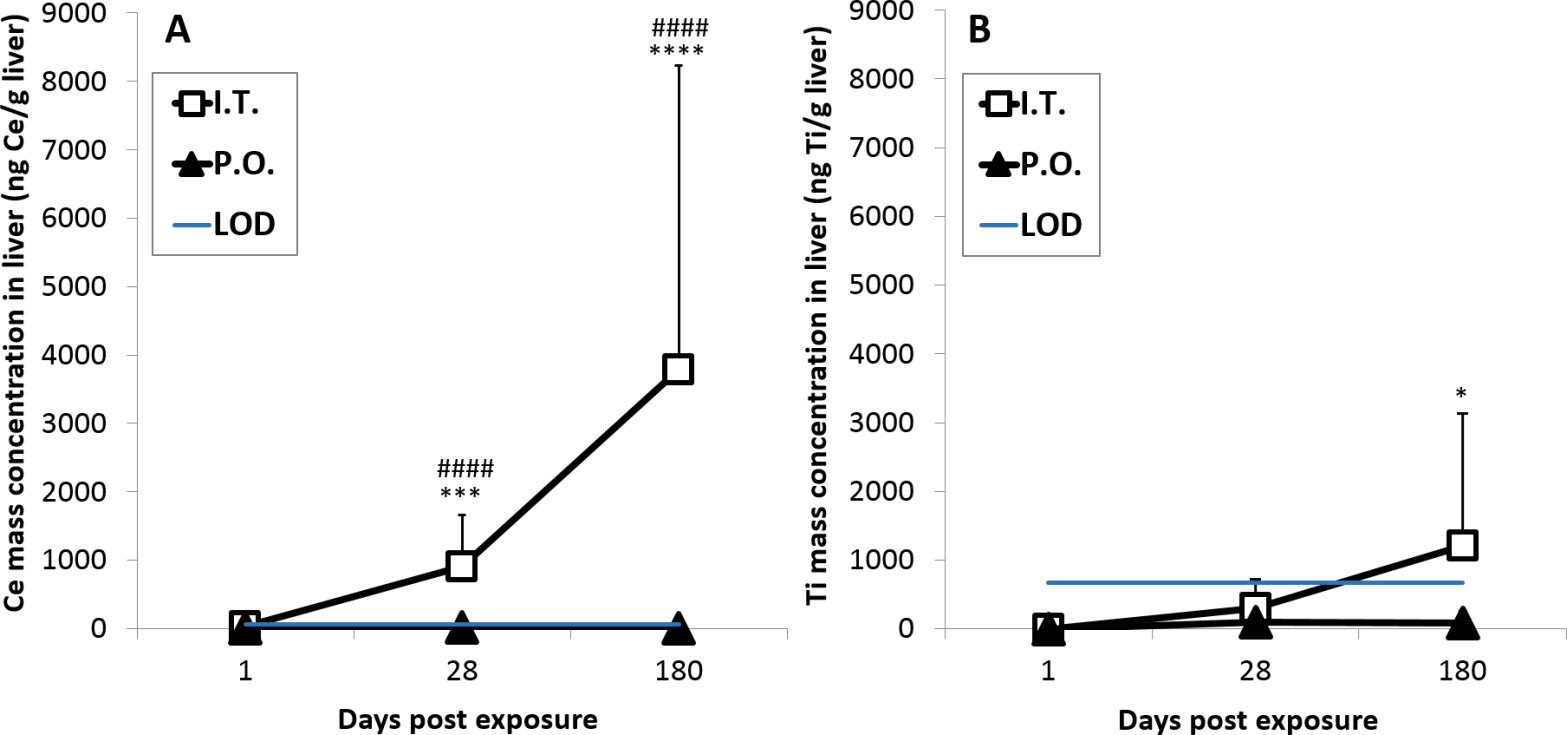
**Mass concentration of Ce (A) and Ti (B) in liver tissue**. The concentrations were measured by ICP-MS following intratracheal instillation and oral gavage of CeO_2_ and TiO_2_ NPs, respectively. Data are presented as mean + SD. An asterisk (*) denotes P ≤ 0.05, (***) P ≤ 0.001, (****) P < 0.0001 of Ce or Ti mass concentration in exposed groups compared to vehicle controls. Hashtags (###) denote P < 0.001 and (####) P < 0.0001 of Ce mass concentration in intratracheally exposed group (IT) compared to orally exposed groups (PO). LOD–Limit of detection.

**Fig 6:**
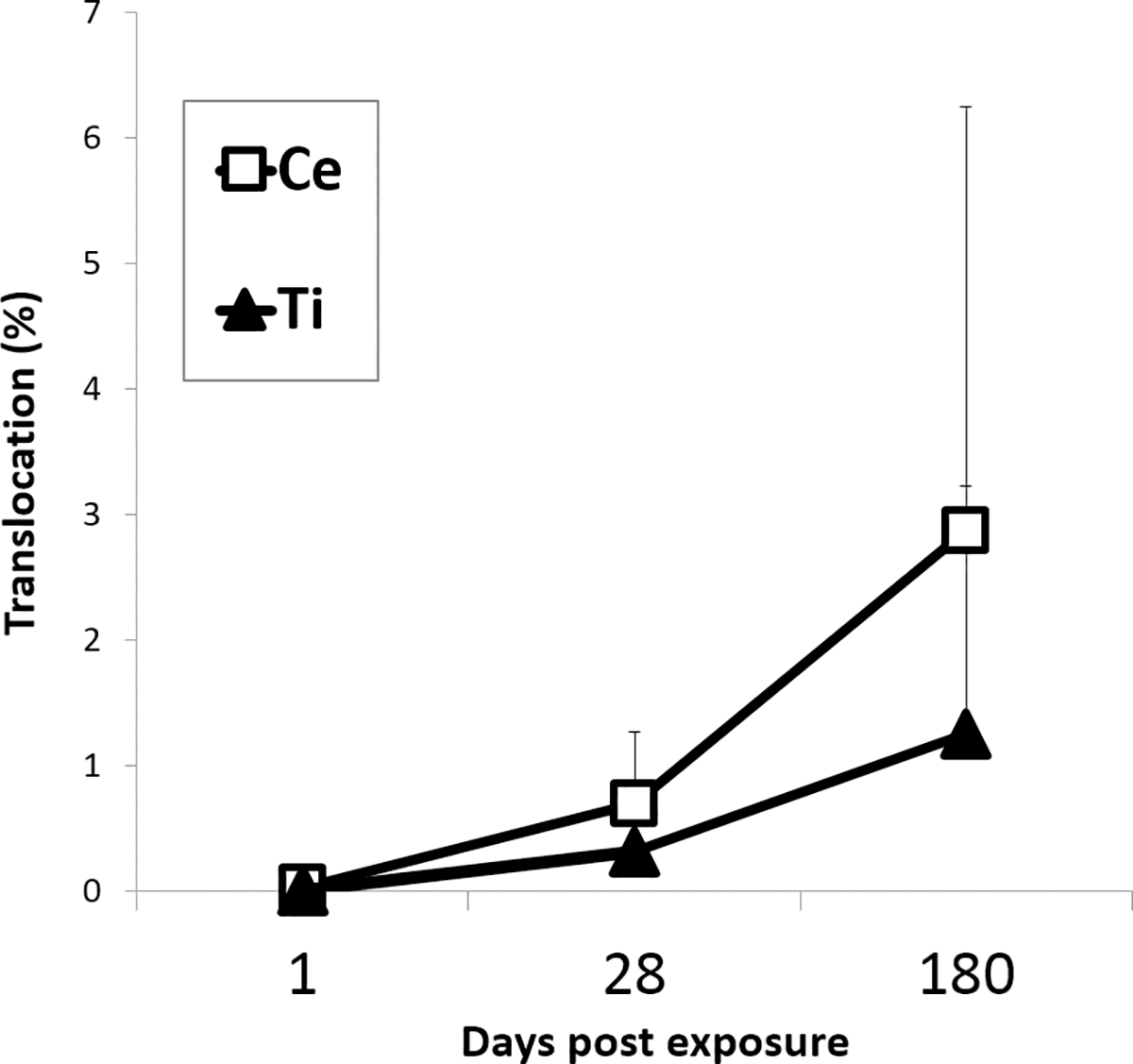
Mass percentage (%) of the total pulmonary dose of Ce or Ti in the liver 1 day, 28 days and 180 days after intratracheal instillation. The calculation was based on a dose of 162 μg CeO_2_ or TiO_2_ NP per lung. Data are presented as mean + SD.

The content of TiO_2_ and CeO_2_ in the liver and lung tissues was estimated by quantifying Ce and Ti mass concentrations by inductively coupled plasma mass spectrometry (ICP-MS). Ce concentrations in the liver were statistically significantly increased 28 and 180 days after intratracheal exposure to CeO_2_ NPs when compared to the vehicle control (P ≤ 0.001 and P ≤ 0.0001, respectively) and when compared to the PO exposed groups (P ≤ 0.0001 and P ≤ 0.0001, respectively) (± A). Ti concentrations in the liver were statistically significantly increased 180 days after intratracheal exposure to TiO_2_ NPs (P ≤ 0.05) when compared to the vehicle control, but no statistically significant differences between IT and PO groups were found at any of the analyzed time points (Fig 5B). The proportion of Ce or Ti that accumulated in the liver 180 days post exposure for CeO_2_ and TiO_2_ was 2.87 ± 3.37% or 1.24 ± 1.98% (mean ± SD), respectively, of the total lung-delivered Ce or Ti dose (based on a NP exposure of 162 μg/lung) (Fig 6). The observed difference in the translocation was not statistically significant. No elevated Ti or Ce concentrations were detected in the livers from orally gavaged mice (Fig 5A and 5B). The Ce and Ti concentrations in liver of vehicle-exposed mice were below the LOD at all assessed time points.

After intratracheal instillation of CeO_2_ NPs, Ce concentrations in lung were statistically significantly increased at all assessed time points (P ≤ 0.001 after day 1, P ≤ 0.0001 after day 28 and P ≤ 0.0001 after day 180) compared to vehicle control (S4 Fig). Ti concentrations in lung after intratracheal instillation of TiO_2_ NPs were also statistically significantly increased at all assessed time points (P ≤ 0.05 after day 1, P ≤ 0.001 after day 28, P ≤ 0.001 after day 180) compared to vehicle control (S4 Fig). At day 1 and day 28 calculated CeO_2_ contents in the lungs were statistically significantly different compared to TiO_2_ contents (P ≤ 0.05 and P ≤ 0.05, respectively) (S4 Fig). The CeO_2_ and TiO_2_ contents were calculated by assuming that all Ce or Ti was present in the form of CeO_2_ or TiO_2_ NPs, respectively. The Ce and Ti concentrations in the lungs of vehicle-exposed mice were below the LOD at all assessed time points.

### Assessment of CeO_2_ NPs size distribution by single particle ICP-MS

**Fig 7:**
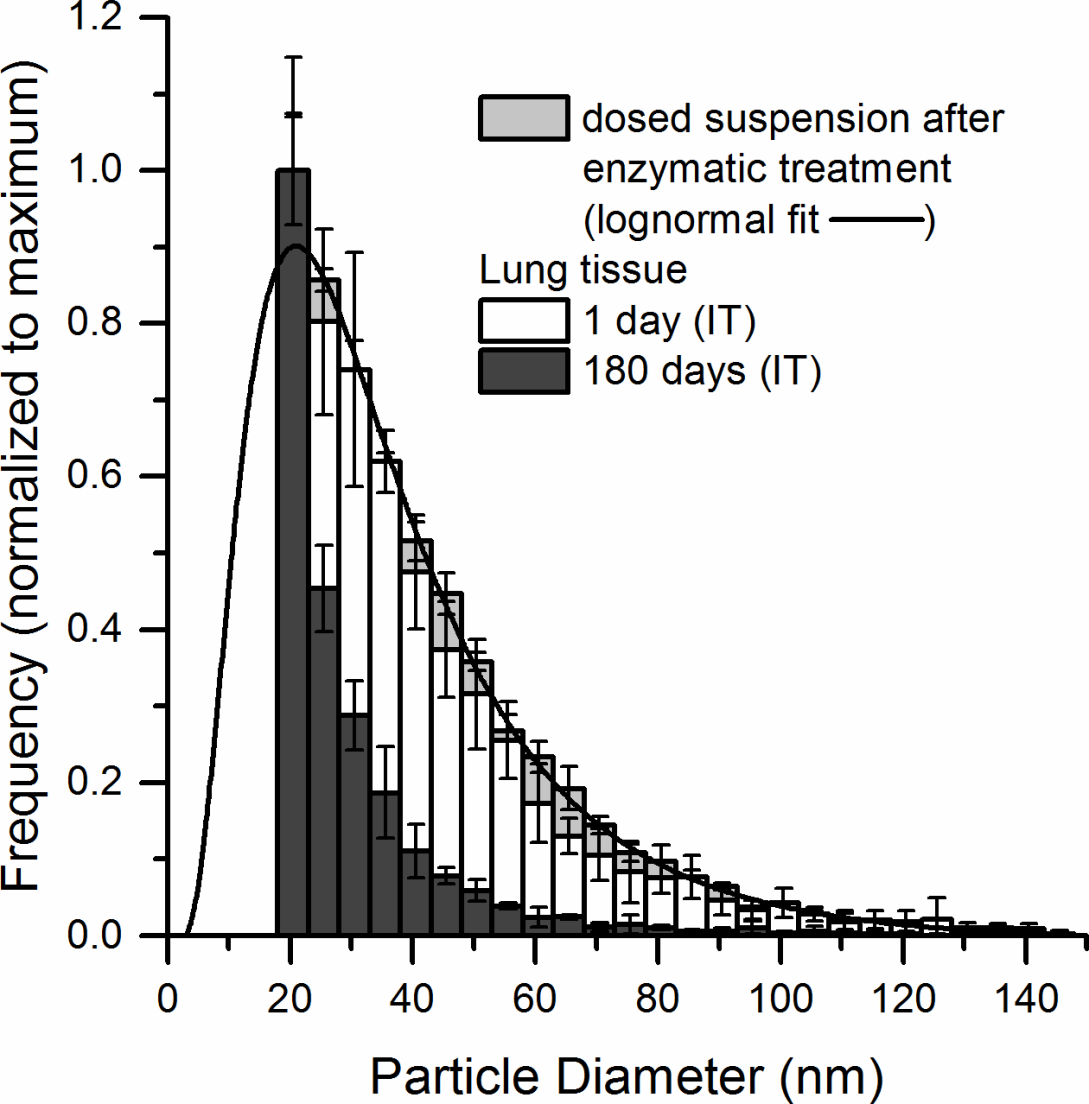
Particle size distribution of CeO_2_ NPs in lung tissue 1 day (N = 6) and 180 days (N = 4) after intratracheal instillation measured by spICP-MS. The distributions were normalized to the most frequently occurring size of the distribution. The particle size distribution of CeO_2_ NP in the dosed suspension after enzymatic treatment is shown for comparison.

The size distributions of CeO_2_ NPs in the liver and lung tissue were assessed by single particle ICP- MS (spICP-MS). The determined sizes were mass-equivalent diameters (diameters of a sphere having the same mass as the studied particle). The spICP-MS technique was used to analyze CeO_2_ NPs in the size range between 18 and 150 nm. An upper size limit of 150 nm was chosen because at higher particle sizes/masses, incomplete particle vaporisation and non-linear detector responses can occur, leading to an underestimation of particle size and mass [54]. Larger NPs (up to 670 nm), most likely aggregates, were detected in all tissue samples but excluded from the quantification. However, on average only 0.6% and at maximum 1.6% of the number of all detected particles was larger than 150 nm. Particles NPs smaller than 18 nm were below the LOD. The size distribution of CeO_2_ NPs in lung tissue (Fig 7) 1 day after pulmonary exposure was similar to the size distribution of the administered particles in 2% serum when subjected to the same enzymatic treatment as the tissue samples. The median particle diameter was 35 nm. 180 days post exposure, the size distribution of pulmonary particles shifted towards smaller sizes with the median particle diameter decreasing from 35 nm (1 day) to 25 nm (180 days). The mass recovery of Ce as 18–150 nm CeO_2_ NPs determined by spICP-MS decreased significantly from 40% ± 28 (1 day, N = 6) to 6% ± 8% (180 days, N = 4) of the total Ce concentration determined by ICP-MS. This was presumably due to the shift of the size distribution to values below the size LOD (Table 3). CeO_2_ NPs frequency in the lung tissue 1 day and 180 days following intratracheal instillation and normalized to the % of recovery of Ce as 18–150 nm CeO_2_ NPs is presented in S5 Fig.

**Table 3:** Mass recovery of Ce in 18–150 nm CeO_2_ particles (determined by spICP-MS) compared to the total Ce mass concentration (determined by ICP-MS) (%) and median particle diameters in the lungs 1 and 180 days post-exposure.

Lungs	Mass recovery of Ce as CeO_2_ NPs compared to total Ce (%)	Median particle diameter (nm)
IT 1 day (N = 6)	40 ± 28	35 ± 3
IT 180 days (N = 4)	6 ± 8	25 ± 1

Values are given as mean ± standard deviation. The presented particle diameters are mass-equivalent diameters.

**Fig 8:**
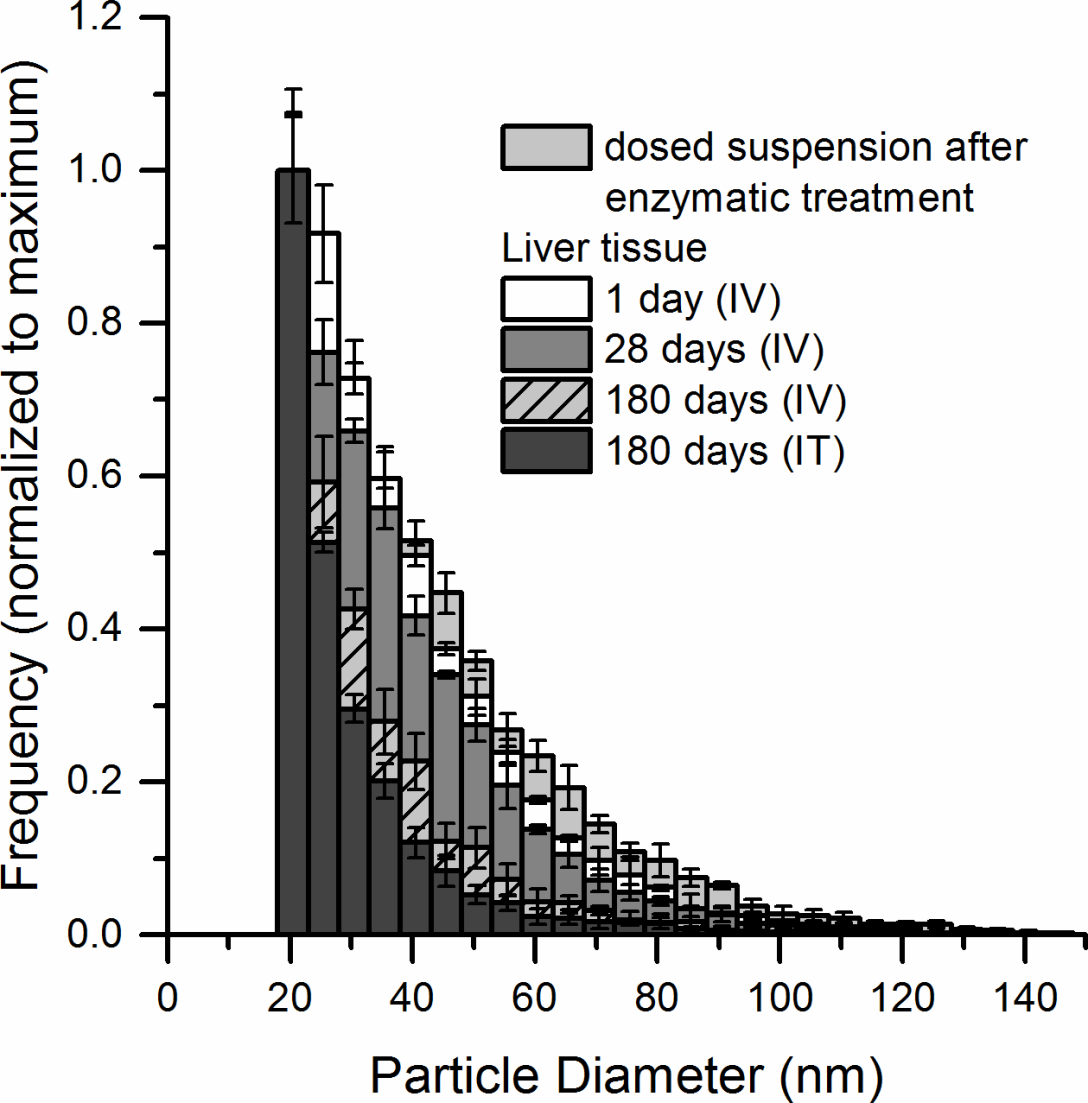
Particle size distribution of CeO_2_ NPs in liver tissue 1 day (N = 3), 28 days (N = 2), 180 days (N = 3) after intravenous injection and 180 days (N = 5) after intratracheal instillation measured by spICP-MS. The size distributions were normalized to the most frequently occurring size of the distribution. The particle size distribution of CeO_2_ NPs in the dosed suspension after enzymatic treatment is shown for comparison.

One day post exposure, the IV dosed particles residing in the liver had the same size distribution as the dosed NPs (Fig 8). The particle size distributions were similar 1 and 28 days post exposure (median diameter 33 nm). Smaller CeO_2_ NPs were detected 180 days post exposure (median diameter 28 nm). Similar to the lung tissues, the mass recovery of Ce in 18–150 nm CeO_2_ NPs (spICP-MS) in comparison to total Ce (ICP-MS) decreased from 66% ± 1 (1 day, N = 3) to 13% ± 5 (180 days, N = 3) (Table 4). CeO_2_ NPs frequency in the liver tissue 1 day, 28 days and 180 days following intravenous injection and 180 days following intratracheal instillation and normalized to % of recovery is presented in S6 Fig.

**Table 4:** Mass recovery of Ce in 18–150 nm CeO_2_ particles (determined by spICP-MS) compared to the total Ce mass concentration (determined by ICP-MS) (%) and median particle diameters in the liver 1, 28 and 180 days post-exposure.

Liver	Mass recovery of Ce as CeO_2_ NPs compared to total Ce (%)	Median particle diameter (nm)
IV 1day (N = 3)	66 ± 1	33 ± 1
IV 28 days (N = 2)	39 ± 4	33 ± 1
IV 180 days (N = 3)	13 ± 5	28 ± 1
IT 180 days (N = 5)	13 ± 7	25 ± 1

Values are given as mean ± standard deviation. The presented particle diameters are mass-equivalent diameters.

The size distribution of the NPs that had translocated from lung to liver had a size distribution that was comparable to the size distribution of the IV dosed particles 180 days post-exposure (Fig 8). Thus, the spICP-MS analyses demonstrated that the size of the CeO_2_ NPs lying above the size LOD of the method decreased with time both in lung and liver tissue.

## Discussion

### Study concept

We studied liver deposition of CeO_2_ and TiO_2_ NPs over time following pulmonary exposure in mice. NPs deposited in the lungs underwent extrapulmonary translocation and liver is one of the major secondary organs for particles sequestration [21,55]. NPs may be cleared from the lungs via mucociliary escalator resulting in subsequent oral exposure or may migrate across the alveolar epithelium, through interstitium and gain access to the systemic circulation. We show that pulmonary deposited CeO_2_ NPs were detected in the liver tissue 28 and 180 days post exposure and TiO_2_ NPs were detected in the liver tissue 180 days post exposure as determined by darkfield imaging and by the quantification of Ce and Ti mass concentration by ICP-MS. Darkfield imaging showed that the foreign material was predominantly found in sinusoids and often in close proximity to small nuclei, probably phagocytized by Kupffer cells. Similarly, a previous study reported that IV dosed gold NPs accumulated in Kupffer cells in the liver [29]. The lack of detection of NPs in the liver following oral gavage by either method indicates that mucociliary clearance and subsequent uptake by the GI tract likely contributes very little to the presence of NPs in the liver after intratracheal instillation. In agreement with this, a recent biodistribution study of radioactively labelled TiO_2_ NPs reported that only 0.05% of the orally dosed TiO_2_ NPs were retained in the body 7 days post-exposure [19]. The same research group estimated that after pulmonary deposition of TiO_2_ NPs at least 80% of the particles that reached the liver had translocated from lung to systemic circulation whereas the remaining 20% may have been taken up from the GI tract [16]. In agreement with this, TiO_2_ NPs have previously been detected in blood smears taken 24 hours after pulmonary exposure [22].

Inhalation is a physiological route for exposure to NPs present in the ambient air, but we decided to use intratracheal instillation since it allowed precise control of the administered dose. In the present study, we exposed animals via three different routes. Therefore, it was important to deliver the same dose regardless the administration method in order to be able to compare results from different groups. The dose used in the present study is relevant for the occupational exposure and corresponds to the pulmonary deposition occurring during thirteen 8-h working days at the Danish occupational exposure limit of 10 mg/m^3^ for TiO_2_, assuming the same particle size distribution during aerosolization as that previously found for similarly sized TiO_2_ NPs [45]. The TiO_2_ and CeO_2_ NPs were dosed by intratracheal instillation as a model of inhaled particles. We used a previously established dispersion protocol to obtain stable NP aggregates and agglomerates. NPs also form aggregates and agglomerates in air [13,45], and thus, we believe that instillation can be used as a model of inhalation even though the dose rate is very different. We used 2% serum in nanopure water as vehicle. The vehicle was chosen based on the ability to form stable dispersions of small NP agglomerates, thus allowing similar and correct dosing of all dosed animals. The spICP-MS analysis of the doses suspension before and after enzymatic digestions confirms the agglomeration of NPs probably due to the presence of the serum proteins (and which are then hydrolyzed by the protease). After enzymatic digestion, primary particles and aggregates of very few particles must dominate (median diameter 35 nm by spICP-MS vs. primary particle sizes of 13±12 nm). We have previously shown that TiO_2_ NP-induced pulmonary inflammation was similar using pure water or 2% serum as vehicle, indicating that the presence of serum did not influence the observed inflammatory response [43]. Furthermore, inflammation was shown to depend on deposited surface area independently of the agglomerate size of the dosed particles. The rates of translocation of TiO_2_ NPs appeared comparable in the present study using intratracheal instillation and in a previously published inhalation study [45]. In the previous study, mice inhaled TiO_2_ NP (diameter 20 nm) for 11 consecutive days. Five days after end of exposure, the lung concentration of Ti was 38 mg/kg corresponding to 38 000 ng/g [54], thus very similar to the Ti concentrations found in lung tissue in the current study (S4 Fig). In the inhalation study, the Ti content in liver tissue was 0.5 mg/kg, corresponding to 500 ng/g, 26–27 days post-exposure. This is very similar to the findings in the current study, where the average Ti content in the liver 28 days post-exposure was 542 ± 308 ng/g liver tissue (Fig 5B).

### Extrapulmonary translocation of NPs

In the present study 180 days following pulmonary exposure, 2.87 ± 3.37% of the total lung-delivered dose was found in the liver of mice intratracheally instilled with CeO_2_ NPs while 1.24 ± 1.98% of the total TiO_2_ dose translocated to the liver. The observed difference in the amount of NPs found in the liver was not statistically significant despite their difference in solubility [56] and their difference in shape (cubic in the case of CeO_2_ NPs and rod in the case of TiO_2_ NPs).

Most studies [23,25,26,45,57] reported low rates (below 1%) of translocation of pulmonary deposited insoluble NPs to secondary tissues [23]. The higher rates of translocation in the current study might be related to the relatively long follow-up time of 180 days.

Rapid and significant translocation of inhaled ultrafine ^13^C particles (count median diameter of 20–29 nm) into systemic circulation followed by subsequent deposition in liver was observed [21]. Fisher 344 rats were exposed by 6 h whole-body inhalation to 80 or 180 μg/m^3^ of ^13^C NPs and liver burden was monitored up to 24 h following exposure. ^13^C NPs were detected in the liver as early as 0.5 h post-inhalation. However, in our study we found no translocation of particles to the liver 24 h following exposure, but this may be caused by a higher LOD in our method.

### Absorption of NPs from the GIT

We found no absorption from the digestive tract neither of CeO_2_ nor of TiO_2_ following oral gavage. These findings are consistent with other studies which have shown that NPs pass through the GIT with no or negligible absorption and are eliminated rapidly via feces [9,12,15,18,19,25,58,59].

### In vivo-induced size transformation of CeO_2_ NPs

We have observed that the median mass-equivalent particle diameter decreased for the CeO_2_ NPs both in the liver and lung tissue 180 days post exposure as compared to 1 day post exposure. A shift towards smaller particle size of IV-administered CeO_2_ NPs and increased reactive surface area has been previously demonstrated and could be a possible explanation for our observation [40]. The *in vivo* induced size transformation was observed in liver 90 days after exposure resulting in formation of very small, 1–3 nm, ultrafine crystallites (called second-generation ceria particles). These small particles cannot be detected by spICP-MS. Here, we report shifts in the size distribution (based on mass-equivalent diameters) of CeO_2_ NPs both in liver and lung tissues. As a possible mechanism for the *in vivo* processing Graham et al. [40] suggested partial dissolution of ceria in the liver promoted by the acidic environment of lysosomes. Since NPs are primarily found in macrophages in both liver and lung tissues [29,60], it is very likely that the dissolution occurs intracellularly in the lysosomal structures.

Further studies should include (cryo)TEM investigations and systematic studies of CeO_2_ NPs in biological relevant media. The observed size distribution (based on mass-equivalent diameters) of CeO_2_ NPs was similar for NPs dosed directly to the liver by IV dosing and the NPs that had translocated from the lung. Particle translocation from lung has been shown to be strongly size dependent [25,28]. However, given the similar size distribution of NPs in the liver following IV and IT exposure, we were unable to determine whether dissolution of the NPs in the lungs resulted in the translocation of the smaller particles or whether the translocated primary particles underwent partial dissolution in the liver.

In the present study, both CeO_2_ NPs that underwent *in vivo* hepatic- and pulmonary-induced changes of the mass-equivalent diameter and virtually insoluble TiO_2_ NPs accumulated in liver over time following pulmonary exposure. Graham and co-workers have previously shown that Ce dissolved from CeO_2_ NPs recrystallized as 1–3 nm NPs, which is in the size range that allows urinary excretion [40]. However, we have no indication that the rate of accumulation of CeO_2_ NPs was lower than the accumulation of TiO_2_ NPs. Thus, the shift in size distribution towards smaller particles that took place both in lung and liver did not lead to reduced liver accumulation.

## Conclusions

The results of the present study indicate that pulmonary deposited CeO_2_ and TiO_2_ NPs are translocated to the liver. The observed particle size distribution of CeO_2_ NPs indicated that CeO_2_ NPs underwent *in vivo* processing in both lung and liver. There is no indication that the partial dissolution in lungs and liver results in increased clearance of CeO_2_ NPs from the organism. The results underscore that liver toxicity is an important endpoint when assessing toxicity of NPs. It is important to establish whether the translocated NPs induce toxic effects in extrapulmonary tissue.

## Supporting information

S1 Fig Particle size distribution of CeO_2_ NPs in the dosed suspension before and after enzymatic treatment measured by spICP-MS. The distributions were normalized to the most frequently occurring size of the distribution.
(TIF)

S2 Fig STEM image of CeO_2_ NPs. (TIF)

S3 Fig Enhanced darkfield microscopy images of H&E stained liver tissue from orally administered mice. (A) From mice that received a control vehicle, (B) 162 μg/animal of CeO_2_ or (C) TiO_2_ NPs 1 day (1) or 180 days (2) post exposure. No apparent foreign material was detected.
(TIF)

S4 Fig Content of Ce and Ti NPs in lung measured by ICP-MS following intratracheal instillation. Data are presented as mean ± SD. Asterisks (**) denote P ≤ 0.01, (***) P ≤ 0.001, (****) P ≤ 0.0001 in exposed groups compared to vehicle controls. Hashtag (#) denotes P ≤ 0.05 and (##) P < 0.01 between Ce and Ti exposed groups.
(TIF)

S5 Fig CeO_2_ nanoparticle frequency in lung tissue 1 day (N = 6) and 180 days (N = 4) after intratracheal instillation measured by spICP-MS and normalized to % m/m recovery of nanoparticles. (TIF)

S6 Fig CeO_2_ nanoparticles frequency in liver tissue 1 day (N = 3), 28 days (N = 2) and 180 days (n = 3) after intravenous injection and 180 days (N = 5) after intratracheal instillation measured by spICP-MS and normalized to % m/m recovery of nanoparticles. (TIF)

S1 Data ICP-MS data for Ti in liver (Figs 5 and 6 in the manuscript). (XLSX)

S2 Data ICP-MS data for Ce in liver (Figs 5 and 6 in the manuscript). (XLSX)

S3 Data Data for spICP-MS diameters (Figs 7 and 8 in the manuscript). (XLSX)
