# Supplementary figures and images for: In vivo-induced size transformation of cerium oxide nanoparticles in both lung and liver does not affect long-term hepatic accumulation following pulmonary exposure
Source: PLoS One. 2018 Aug 20;13(8):e0202477. doi: 10.1371/journal.pone.0202477 (PMC6101382; doi:10.1371/journal.pone.0202477)

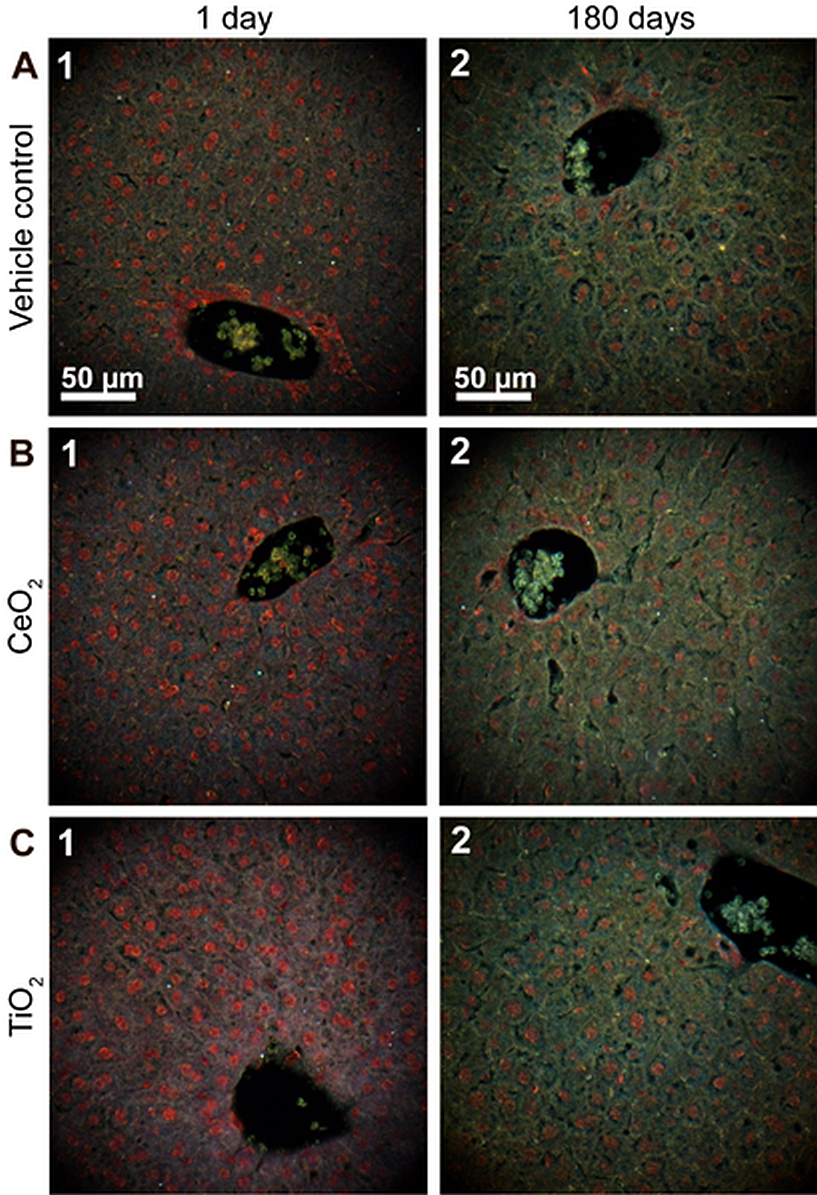

Supplement: S3 Fig — (A) From mice that received a control vehicle, (B) 162 μg/animal of CeO2 or (C) TiO2 NPs 1 day (1) or 180 days (2) post exposure. No apparent foreign material was detected. (TIF) [file pone.0202477.s003.tif]

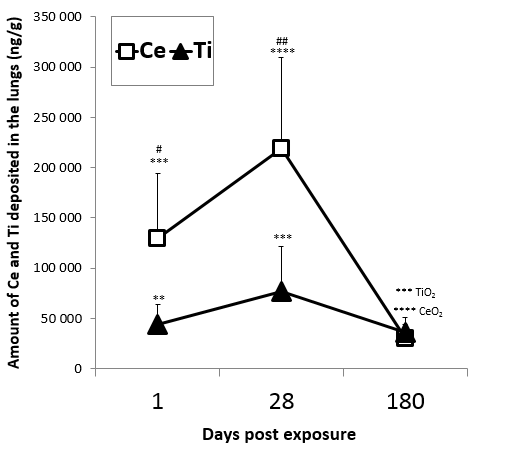

Supplement: S4 Fig — Data are presented as mean ± SD. Asterisks (**) denote P ≤ 0.01, (***) P ≤ 0.001, (****) P ≤ 0.0001 in exposed groups compared to vehicle controls. Hashtag (#) denotes P ≤ 0.05 and (##) P < 0.01 between Ce and Ti exposed groups. (TIF) [file pone.0202477.s004.tif]
